# Immunotoxicological impact and biodistribution assessment of bismuth selenide (Bi_2_Se_3_) nanoparticles following intratracheal instillation in mice

**DOI:** 10.1038/s41598-017-18126-y

**Published:** 2017-12-21

**Authors:** Vani Mishra, Vikas Baranwal, Rohit K. Mishra, Shivesh Sharma, Bholanath Paul, Avinash C. Pandey

**Affiliations:** 1 Nanotechnology Application Centre (NAC), University of Allahabad, Allahabad, 211002 India; 2 NMR Section, SAIF, CSIR-Central Drug Research Institute (CDRI), Lucknow, 226031 India; 3 Centre for Bioresource Innovation and Research (CBIR), Dept. of Microbiology, Swami Vivekanand University, Sagar, 470228 M.P. India; 4 Centre for Medical Diagnostic and Research (CMDR), Motilal Nehru National Institute of Technology (MNNIT), Allahabad, 211004 India; 5 Immunobiology Division, CSIR-Indian Institute of Toxicology Research (IITR), Lucknow, 226001 India

## Abstract

Variously synthesized and fabricated Bi_2_Se_3_ nanoparticles (NPs) have recently been explored for their theranostic properties. Herein, we investigated the long term *in-vivo* biodistribution of Bi_2_Se_3_ NPs and systematically screened its immune-toxic potential over lungs and other secondary organs post intratracheal instillation. X-Ray CT scan and ICP MS results revealed significant particle localization and retention in lungs monitored for 1 h and 6 months time period respectively. Subsequent particle trafficking was observed in liver, the major reticuloendothelial organ followed by gradual but incomplete renal clearance. Pulmonary cytotoxicity was also found to be associated with persistent neutrophilic and ROS generation at all time points following NP exposure. The inflammatory markers along with ROS generation further promoted oxidative stress and exaggerated additional inflammatory pathways leading to cell death. The present study, therefore, raises serious concern about the hazardous effects of Bi_2_Se_3_ NPs and calls for further toxicity assessments through different administration routes and doses as well.

## Introduction

Bismuth selenide nanoparticles (Bi_2_Se_3_ NPs) have started to show a vast potential in biomedical applications including disease diagnosis as well as their treatment^1–3^. Belonging to the class of topological insulators in quantum matter physics they have been observed to possess 3d structure with single Dirac cone^4^. Furthermore, on account of its remarkable thermoelectric, optical and photoelectrical characteristics^5–7^, Bi_2_Se_3_ NPs have attracted much attention of chemists and biophysicists. Bi_2_Se_3_ is made up of Bismuth (Bi), known for its low toxicity and therapeutic implication and Selenium (Se), which in its elemental form is known to play pivotal roles in human immune and enzymatic systems^8,9^. Much recently, because of its high atomic number (Z = 83), high X-ray attenuation coefficient and photothermal conversion efficiency, Bi has become an attractive element for contrast agent in cancer imaging, diagnosis and therapy^9–12^. Since particles at their nanoscale have been reported to incur severe damage to the target organs, their pharmacokinetic behavior, long-term fate and toxicity profiles remain to be the most important concern, before they could be practically translated to the clinics^13–15^.

Lungs are the major organs that communicate with the surroundings and remain the primary target for foreign invasions. These are also the major route of accidental as well as environmental exposures of engineered nanoparticles^16^. Various reports have established that nanoparticles (<100 nm) get deposited throughout the length of the respiratory tracts with 20% concentration in alveolar region and 5% in tracheobronchial region^17^. This deposition pattern is independent of aerodynamic impaction and sedimentation due to their nanosized diameter; they however get deposited by means of diffusion^18^. From deep into alveolar spaces they traffic towards extrapulmonary organs via blood or tracheobronchial lymph nodes^19^; the critical event being the inflammatory responses of acute, sub acute and chronic levels supported by inflammatory cells infiltration and modulation of immune cascade^20^. Nanoparticle trafficking across primary and secondary organs leads to transmittance of physiological signals across membranes affecting basal functioning of various organs. Thus, studying nanoparticle toxicity and immune response over whole body is of extreme importance^21^.

Researchers have started employing functionalized NPs, owing to their easy metabolomics and prolonged circulation time that enhances their permeability and retention in target organs^22,23^ and facilitate their utilization as contrast agents for X-Ray and MRI imaging^10,24^. Attributing to their high hydrophobic surface and non-biodegradable nature CNTs have raised serious concern of reduced biocomaptibilty as well as enhanced chronic cytotoxicity, thereby limiting their usage in biomedical applications^25^. Both single and multiple wall CNTs have been found to induce cytotoxicity through membrane damage, DNA damage, oxidative stress, alterations in mitochondrial activities and protein synthesis ultimately converging to necrosis and apoptosis^26,27^. Furthermore, nanoparticles of other classes including Graphene oxide and other metal oxides have also shown similar pattern of toxicity profiles^28,29^. Albeit long term biokinetic studies of PVP functionalized Bi_2_Se_3 _nanoplates have been found to exhibit low toxicity when administered intraperitoneally, their concomitant translocation and accumulation to secondary organs have also been well documented^21^. In line with this requirement, Bi_2_Se_3 _NPs have not been evaluated for their immune-toxic profile over lungs following intratracheal instillation. Herein, we report pulmonary toxicity and biodistribution of Bi_2_Se_3 _NPs in Balb/c mice for 6 months. In order to evaluate *in-vivo* biodistribution within 60 min, we employed X-Ray CT scan imaging, while long term biokinetics was assessed through ICP-MS. Toxicity profile of lungs and other secondary organs was measured through BALF and serum biochemistry as well as histopathology. Since the NP-mediated toxicity is mainly driven and dominated by immune responses, we evaluated cell damage in terms of cell permeability, inflammatory markers and oxidative stress.

## Results and Discussion

### Synthesis and Characterization of NPs

**Figure 1:**
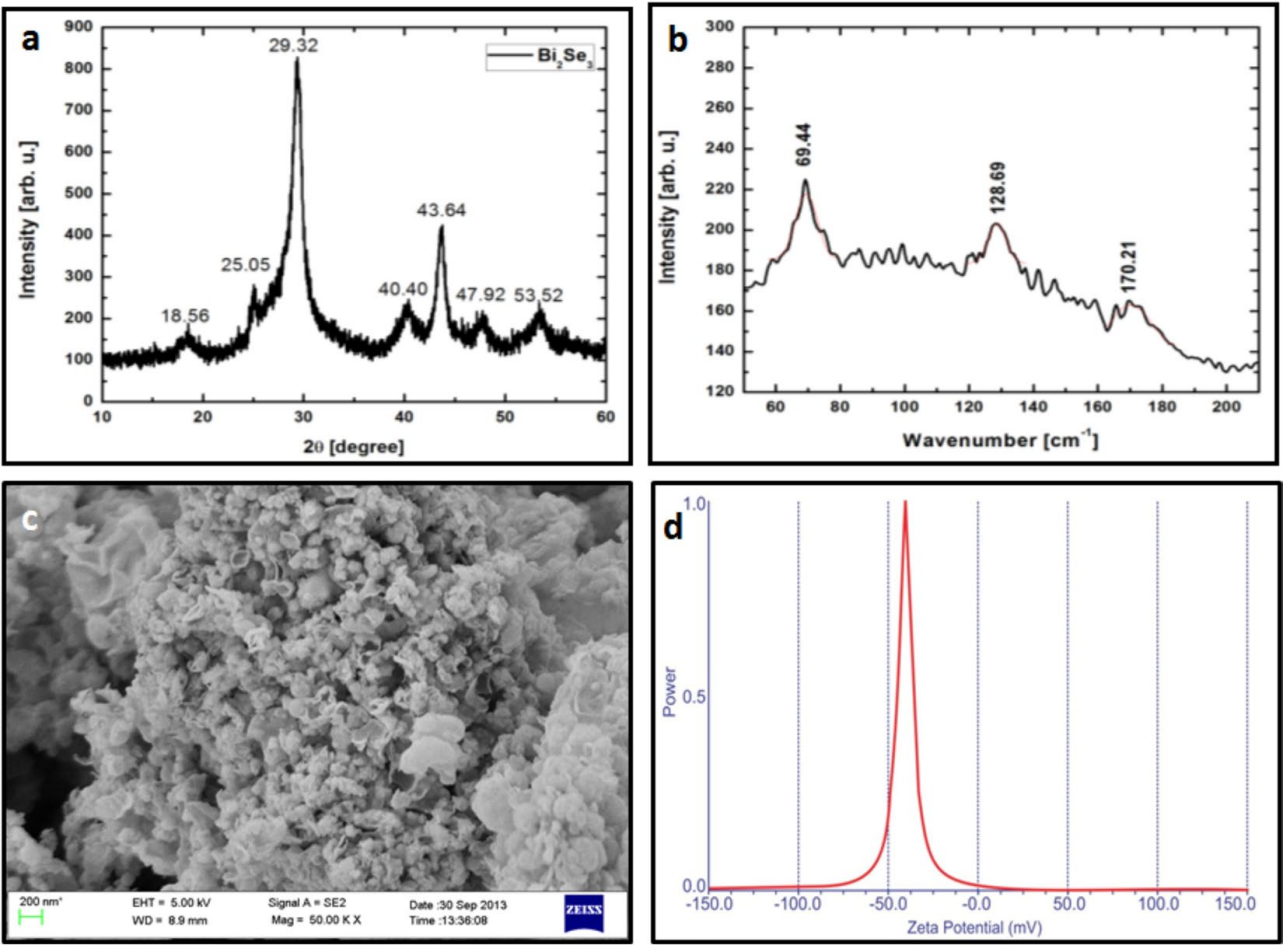
Selected Nanoparticles Characterization. (**a**) XRD pattern of as-prepared Bi_2_Se_3_ NPs (**b**) Raman Spectroscopy (**c**) SEM image (**d**) Particle stabilization was measured through Zeta Potential.

Bi_2_Se_3_ NPs were prepared and characterized using X-ray diffraction, scanning electron microscopy and Raman spectroscopy. A typical X-ray diffraction pattern is shown in Fig. 1a. XRD spectra show broad peaks at 18.56, 25.05, 29.32, 40.40, 43.64, 47.92, 53.52° corresponding to the planes (006), (101), (015), (101), (110), (116), and (205) respectively. These peaks are matched with JCPDS card no. 33-0214 with a rhombohedral structure and space group R-3m. The strong diffraction peak at 29.32° represents the prominent growth of NPs along [015] direction. No other crystalline phase was observed signifying the high purity and crystallinity of the Bi_2_Se_3 _NPs. The average size of the crystallites was estimated by Scherrer’s formula and found to be ~8 nm. Figure 1b shows the Raman spectra of the as synthesized Bi_2_Se_3 _NPs in which three different Raman peaks corresponding to three resonance modes are clearly evident. The three peaks located at 72, 131, 174 cm^−1^ are assigned to the A^1^_1g_, E^2^_g_, A^2^_1g_ vibrational modes, respectively^30–32^. The high background signal is caused by the broad photoluminescence contribution. This explains the absorption and the severe reduction in the intensity of different phonon modes^33^. Figure 1c shows the SEM image of as prepared Bi_2_Se_3_ NPs. The SEM image shows flake like nanostructures of Bi_2_Se_3_ with an average size of 70 nm. The growth of these nanoflakes may be attributed to the layered nature of Bi_2_Se_3_ structure^34^. Further Zeta potential of NPs in pure water was estimated to be −34.28 mV (Fig. 1d). The high negative zeta potential values suggest that the NPs are charge stabilized^35^.

### Biotrafficking of Bi_2_Se_3_ NPs

**Figure 2:**
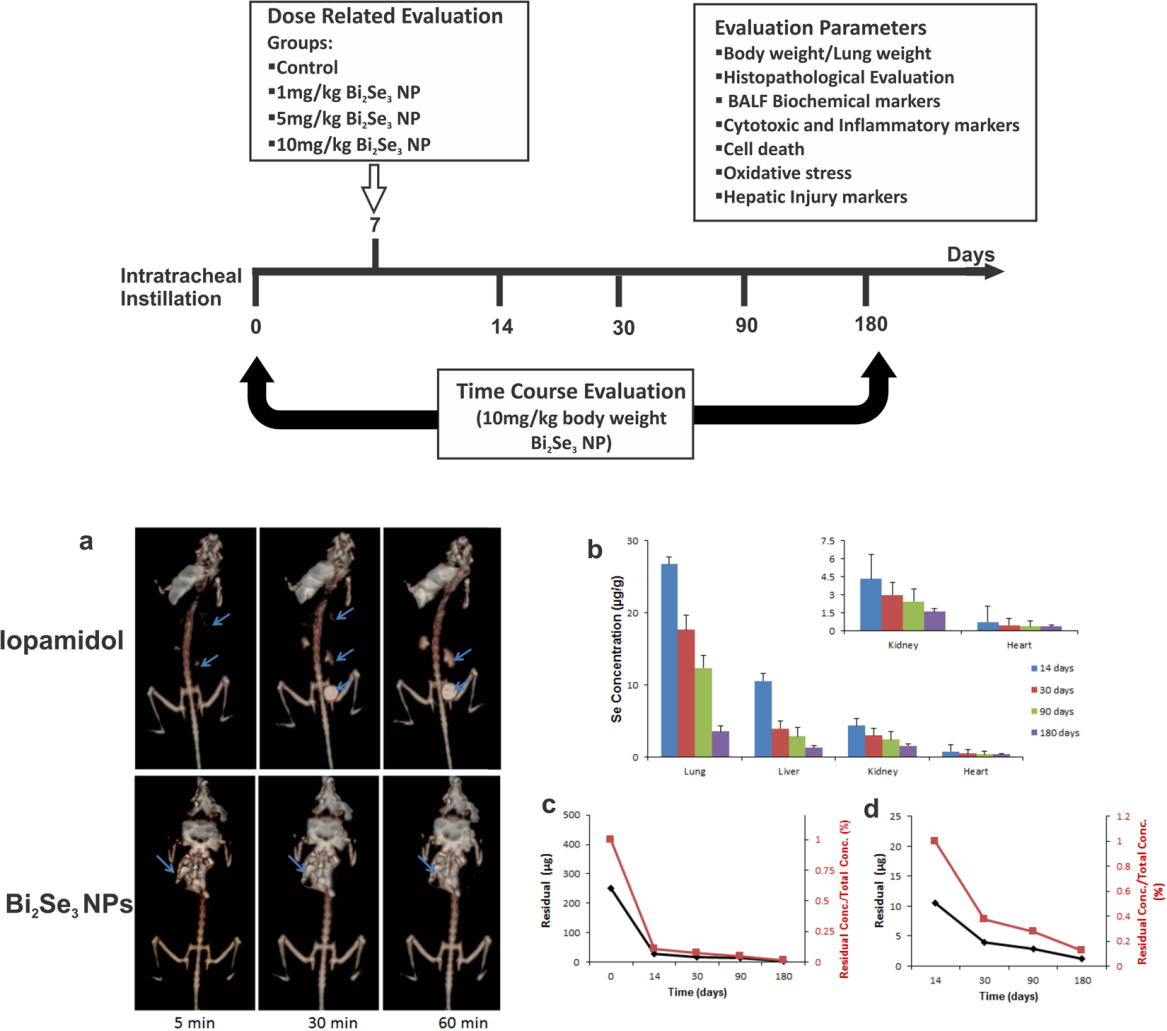
Schematic representation and *in-vivo* biodistribution ad clearance of Bi_2_Se_3_ NPs. Upper panel represents general experimental design which includes a dose related (Control, 1 mg/kg, 5 mg/kg and 10 mg/kg body weight) as well as time course evaluation (Control, day 14, 30, 90 and 180) of Bi_2_Se_3_ NP induced toxicity. The control group did not receive any NP doses. Lower Panel (**a**) X-Ray CT images at 0, 30 and 60 min time points after intratracheal instillation of NPs or Iopamidol in mice following anaesthesia. (**b**) Se concentration measured through ICP MS at 0, 14, 30, 90 and 180 days post intratracheal instillation. Values are represented as Mean ± SEM (N = 5 mice/group) (**c**) and (**d**) Residual particle concentration in lungs and liver representing time dependent clearance.

The biodistribution pattern of Bi_2_Se_3 _NPs in mouse model has been studied through two methods, one being X Ray Computed Tomography and the other by ICP MS. Iopamidol, an established and widely used contrast agent^36^ was employed as control to study *in-vivo* trafficking of Bi_2_Se_3 _NPs. The X Ray CT image (Fig. 2a) displayed high contrast in lungs immediately after intratracheal instillation that persisted for 60 min time span indicative of particle retention in lungs. No particle trafficking was observed towards extrapulmonary organs for about 60 min as no contrast was observed in kidneys or urinary bladder. Similar trend of particle retention was observed with intratracheally instilled nano graphene oxide sheets monitored through SPECT that report penetration of small sized particles to blood by alveolar capillary barrier and eliminated through renal route^28^. However, during similar time frame, Iopamidol migrated towards kidney and urinary bladder. As time elapsed, contrast faded from lungs and gradually increased in kidneys and urinary bladder indicating exclusion of contrast agent from the body by urinary route.

Inhalation is an established mode of particle administration, providing large surface area for particle accumulation in alveolar region, more precisely in alveolar type I epithelial cells^37,38^. Upon retention in lungs, particles penetrate into the deep air spaces of alveoli where they are readily taken up by lung epithelial cells and fibroblasts. The lymphatic drainage system facilitates the translocation of the retained particles to secondary organs via blood capillaries^39^. ICP-MS further evaluated long term biokinetics of the particles in different organs at 14, 30, 90 and 180 day time interval (Fig. 2b). No mortality was observed in the treated groups and no sign of toxicity was recorded in whole body weight and organ weight specifically. Particle localization at all time points was significantly concentrated in lungs; however, time dependent decrease in particle concentration was observed. About 37.76 µg/g of particles were retained in lungs till day 14, which subsided to 29.69 µg/g on day 30, 17.4 µg/g on day 90 and subsequently to 8.56 µg/g on day 180 post intratracheal instillation. Residual particle retention (%) in lung from day 14 to day 180 subsided from 15.10–3.4% (Fig. 2c). In spite significant clearance considerable amount of Bi_2_Se_3 _NPs were found to accumulate in lungs for as long as 180 days, which is similar to the previous findings by other groups^40^.

Further interaction with the macrophages and other resident cells of the lungs, facilitates the relocation of the particles beyond epithelium into secondary organs^41,42^. In this study we continued to evaluate biotrafficking of Bi_2_Se_3 _NPs to secondary organs including liver, kidneys and heart during 180 day time period following intratracheal instillation. On day 14, 10.57 µg/g of Bi_2_Se_3 _NPs was recovered from liver. On day 90 and 180, normalized particle accumulation was reduced by ~4 and 1.2% respectively (Fig. 2d). Similar trend was observed in case of kidney, thereby implying significant particle retention in kidneys and liver. Notably, no particles were detected in blood at all time points, indicating particle concentration below detection limit. Rapid clearance of the particles form blood could also be attributed to reticuloendothelial system (RES), where particle uptake is facilitated by macrophages sequestering into secondary organs^43^. Particle translocation and accumulation has also been observed in liver, a major target organ of RES, indicating systemic circulation of particles. Our results corroborated with the findings previously reported^2^. ICP-MS results revealed time dependent decrease in particle accumulation in lungs from 14 day to 180 day, whereas a considerable similar particle concentration was found in liver on 30 day and 90 day, clearly indicating retention of Bi_2_Se_3_ NPs in RES^29^.

**Figure 3:**
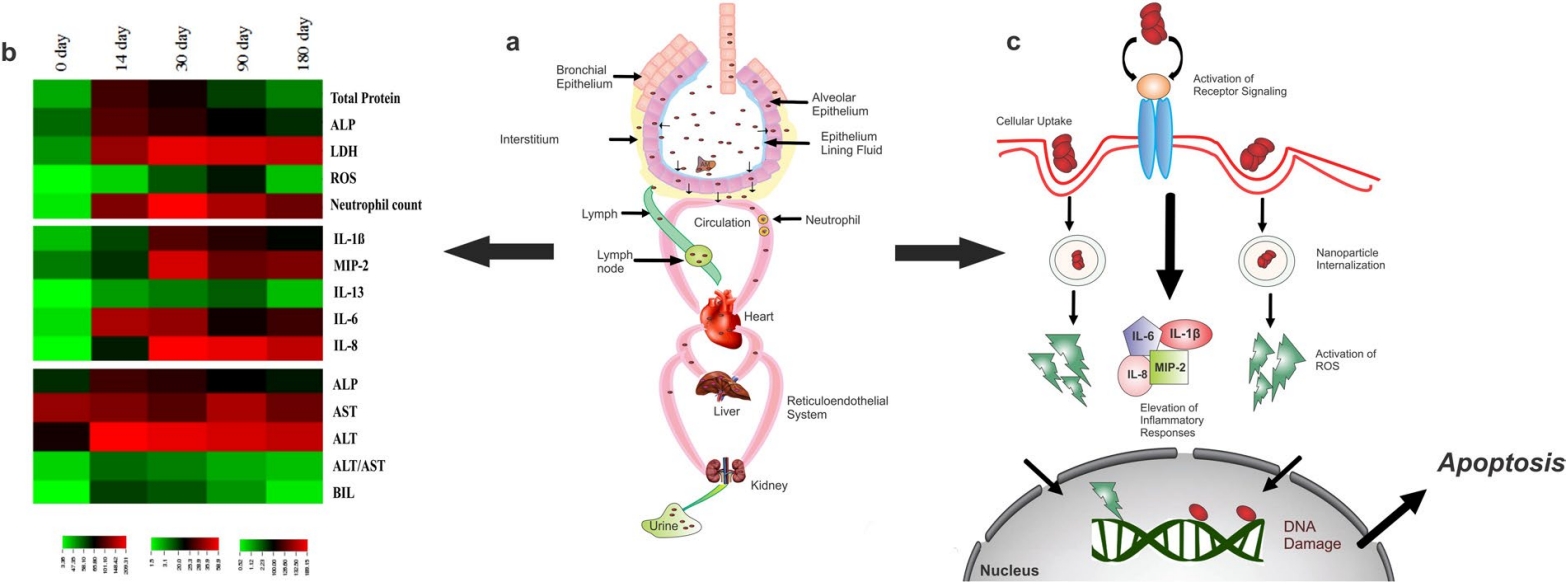
Schematic representation of (**a**) nanoparticle translocation and accumulation across lung epithelium towards liver (reticuloendothelial system) and subsequent clearance through renal route. (**b**) Clustered Image Map of immunotoxic assay suit. The biochemical and the inflammatory markers were represented as heat map in terms of their severity for comparative analysis at different time points. Assay suits are represented in red color indicating the maximum while green indicating the minimum value of the data set corresponding to each parameter. (**c**) Proposed pathway representing upregulation of inflammatory mediators upon nanoparticle internalization which subsequently results in enhanced oxidative stress and further ROS mediated cell death.

Figure 3a explains the systematic trafficking of ~8 nm Bi_2_Se_3_ NPs administered intratracheally with maximum retention in lungs, the primary target organ and then in liver, the secondary target organ and a major component of RES; kidney, however, did not suffer with major toxicity concerns. It has already been document that NPs < 5.5 nm can be cleared by the kidney, while large NPs > 50 nm may be partially cleared by the liver with the function of time^21,44^ Zhang *et al*. (2014)^2^ reported increased PVP coated Bi_2_Se_3_ NPs accumulation in kidney after 90 days, which bears disparity with our findings. This discrepancy could be attributed to PVP coating over the NPs that could enhance blood circulation time and thus promoting particle absorption by kidney^27,45^. Also longer persistence of NPs in blood could induce the formation of biomolecular corona around the NPs, thereby further increasing their circulation time in blood^44,46^.

### Pulmonary Toxicity induced by Bi_2_Se_3_ NPs

**Figure 4:**
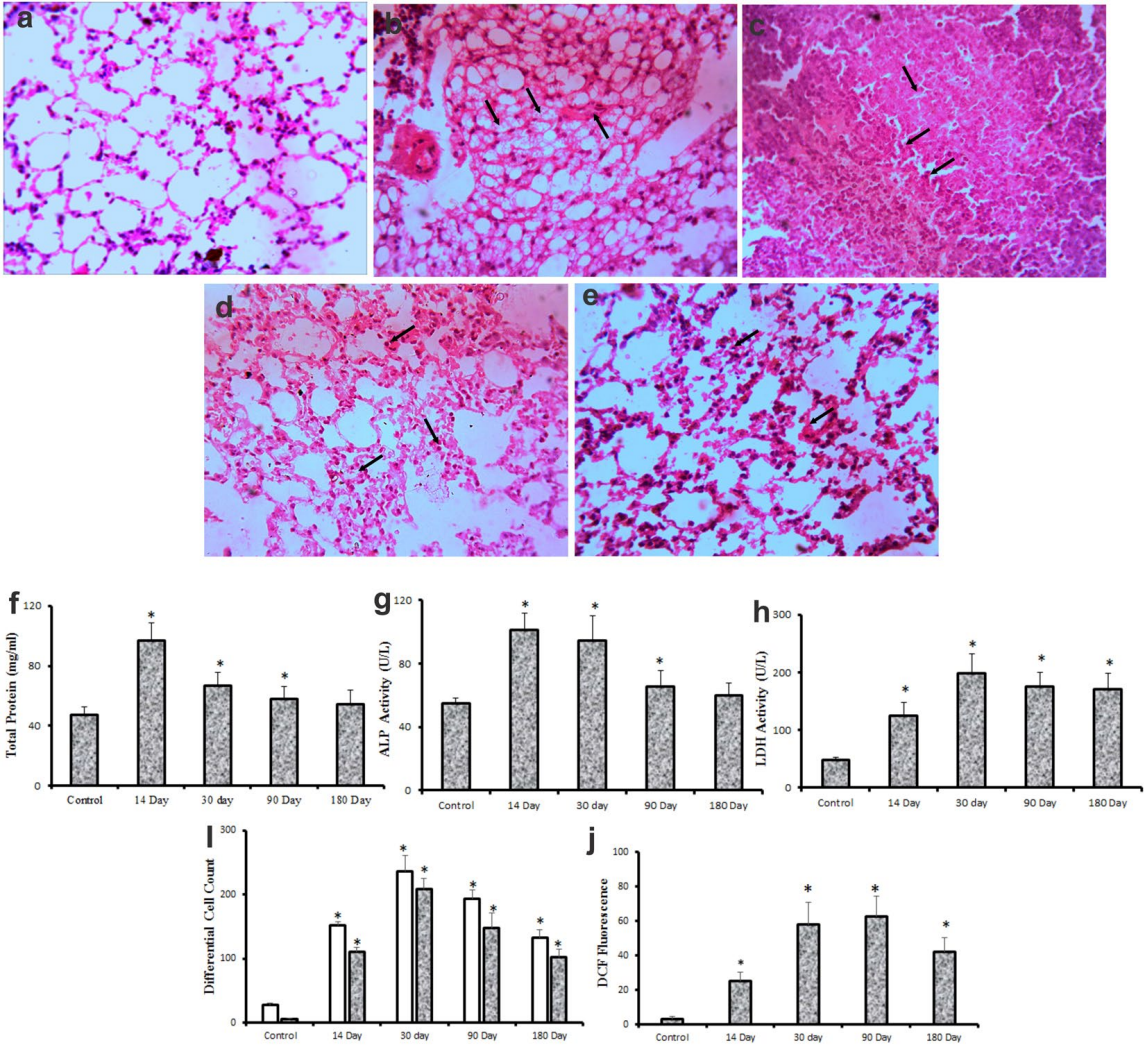
Toxicity profile assessment in lungs induced by Bi_2_Se_3_ NPs at different time points. (**a–e**) Photomicrograph of representative section of lungs excised from (**a**) control and treated groups on (**b**) day 14 (**c**) 30 day (**d**) 90 day and (**e**) 180 day. Images reveal severe lung damage in treated groups due to infiltration of inflammatory cells, capillary dilation and accumulation of interstitial connective tissues. (**f–j**) Time course evaluation of biochemical markers of lung damage and cytotoxicity in response to Bi_2_Se_3_ NPs exposure. BALF supernatant from treated and untreated lungs were obtained and analyzed for (**f**) Total Protein (**g**) ALP (**h**) LDH (**i**) Neutrophil count. Lung homogenates prepared from control and exposed groups were used to measure (**j**) ROS generation. All data are represented as the mean ± SEM (N = 5 mice/group) p < 0.05 compared to control group.

*In vivo* dose dependent pulmonary toxicity was evaluated at different doses (1, 5, 10 mg/kg Bi_2_Se_3_ NPs). Deviations in organ and lung weight were observed in treated groups as compared to control (Fig. S1). Similarly histopathological evaluations also revealed dose dependent edema, inflammatory infiltrates and thickening of alveolar septa (Fig. S2). No mortality was, however observed in any of the treated groups. We also investigated the toxicological impact of highest dose of Bi_2_Se_3 _NPs (10 mg/kg) over different organs of mouse administered through intratracheal instillation. The light micrographs images revealed moderate inflammation in lungs on day 14 with thickening of alveolar septa and damage in alveolar borders. Thereafter, on days 30, 90 and 180 severe lung damage was observed indicating progressive fibrosis (Fig. 4a–e). Inflammatory cell infiltrates were also observed in bronchial lumen, primarily due to capillary dilation or accumulation of interstitial connective tissues. Arrows indicate Bi_2_Se_3_ aggregates in alveolar macrophages of all treated groups over control; particle congestion leading to alveolar oedema and hemorrhage. This observation suggests alveolar macrophage mediated particle clearance via mucocilliary action. It could also be concluded that degree of lung pathology is a function of exposure time; however, individual immunity has to be taken into account.

**Figure 5:**
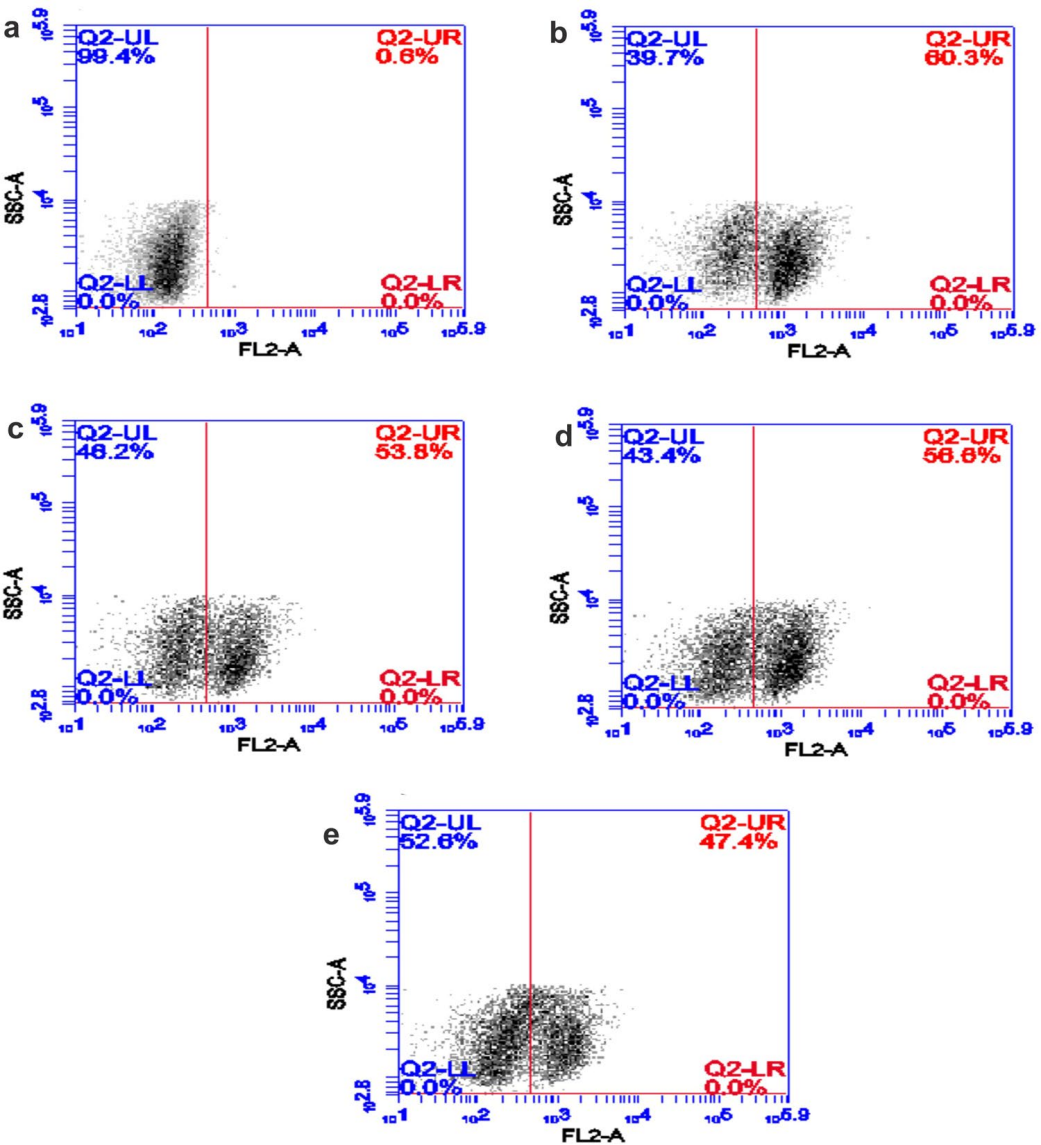
Measurement of cell death by PI uptake upon Bi_2_Se_3_ NPs exposure. BALF cells from (**a**) control and treated groups of different time points (**b**) 14 day (**c**) 30 day (**d**) 90 day and (**e**) 180 days were stained with PI and visualized using flow cytometer. Density plots showing percentage live cells in upper left (UL) quadrant while percentage dead cell in upper right (UR) quadrant.

In order to gain a comprehensive understanding about the NP impact *in-vivo*, blood and BALF were collected from the animals to evaluate changes in biochemical profiles. BALF cells were stained with Propidium Iodide (PI) to study NP-mediated cell death through flowcytometry (Fig. 5a–e). PI internalization was observed to be maximum on day 14 showing ~60% cell death followed by about 55% cell death on day 30 and 90. The cytotoxicity reduced to ~47% on day 180 subsequently revealing lowered NP-mediated toxicity. The changes in total protein and LDH were measured to evaluate damage in alveolar-capillary barrier and general cytotoxicity^47^, while increased ALP activity in BALF indicates increased secretory activity of alveolar type II cells, or injury of these cells^48^. Furthermore, ROS and neutrophil count were estimated to study the oxidative stress and inflammatory response in cells^49^. Consistent upregulation of all the parameters was observed in all treated groups. Significant elevation (p < 0.05) in LDH was detected throughout the exposure duration with maximum activity on 30 day (Fig. 4h), emphasizing long term persistence of Bi_2_Se_3_ NPs induced pulmonary injury; however, Total protein and ALP levels (Fig. 4f and g) on 180 day became comparable to the control group indicating reversal of cytotoxicity in particular treated group. Bi_2_Se_3_ NPs exposure further resulted in significant increase in the total number of cells recovered in BALF when compared to untreated group. Also, significant upregulation of neutrophils (Fig. 4i), a characteristic marker of initial inflammatory stage was observed at all-time points with maximum count of about 209.3 ± 16.31 on day 30. On day 180 the neutrophil counts subsided to 102.65 ± 12.1 but maintaining the significance over the control group. Persistent occurrence of neutrophil over NP administration indicated towards continuous neutrophil infiltration and progressive lung injury.

We further evaluated the level of Reactive Oxygen Species (ROS) to know whether Bi_2_Se_3_ NPs could induce oxidative stress in the lungs. A time dependent increase in ROS level (Fig. 4j) was observed for day 14 (~7.5 fold), 30 (~17.3 fold) and 90 (~18.6 folds) than the control group; the level, however declined on day 180 to ~12.5 folds. Significant increase in LDH and total protein could imply damage to membrane integrity leading to neutrophil infiltration^50^, characterized by respiratory burst and substantiate through increased ROS production^51^. Previous published reports also confirm NP-mediated pulmonary toxicity pertaining to increased cell death, LDH, ALP and total protein activity in BALF^20,52,53^.

**Figure 6:**
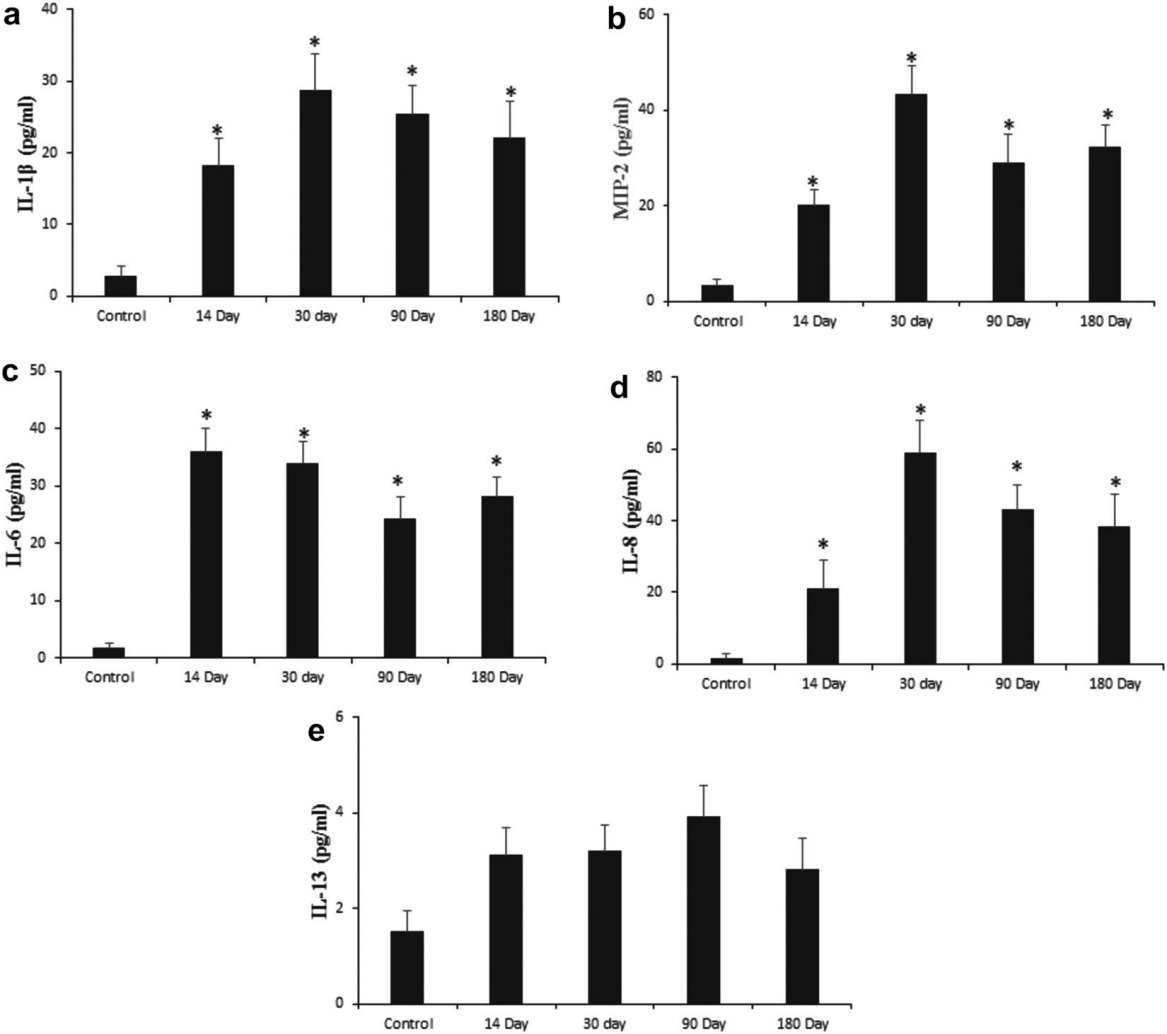
Time course evaluation of inflammatory mediators in response to Bi_2_Se_3_ NPs exposure. BALF supernatant from treated and untreated lungs were obtained and analyzed for (**a**) IL-1β, (**b**) MIP-2, (**c**) IL-6, (**d**) IL-8 and (**e**) IL-13. All data are represented as the mean ± SEM (N = 5 mice/group) p < 0.05 compared to control group.

Oxidative stress via ROS generation following NP administration since long has been considered as a major concern for cellular damage that further induce various pro-inflammatory pathways^54^. In consideration with this fact, we evaluated the cytokine profiles underlying NP-induced inflammation. We measured the inflammatory markers (Fig. 6a–e) for cellular damage via oxidative stress (IL-6 and IL-8), neutrophilic (IL-1β, MIP-2), eosinophilic (IL-13 and eotaxin) and lymphocytic inflammation (IFN-γ) in BALF. On day 14 post dose administration, both IL-1β and MIP-2 increased ~ 6 folds; IL-6 and IL-8 also recorded an increase of ~22 and 14 folds respectively; IL-13 however did not show any significant change. Eotaxin and IFN-γ were below detection limit (not shown). All these changes were recorded over the background of control group. Proceeding towards further time points we observed similar expression profiles of the cytokines.

Considering the immunological footprints, we concluded that Bi_2_Se_3_ NPs induced persistent neutrophilic/mildly cytotoxic inflammation associated with increased expression of IL-1β, MIP-2 and IL-6, corroborating with increased LDH level in BALF and neutrophil counts in blood; however, eosinophilic or lymphocytic kind of inflammation was not observed as the associated markers IL-13 and eotaxin for the former and IFN-γ for the later were downregulated. Our findings are similar to the previous observations made with Carbon Nano tubes and metal oxide NPs like CeO_2_ and NiO, ultrafine ZnO as well as TiO_2_ that have been found to induce neutrophilic/cytotoxic inflammation followed by an increase in IL-6, TNF-α, IL-1β. MIP-2 and IL-8^55–57^.

### Toxicological Impacts of Bi_2_Se_3 _NPs in extrapulmonary organs

**Figure 7:**
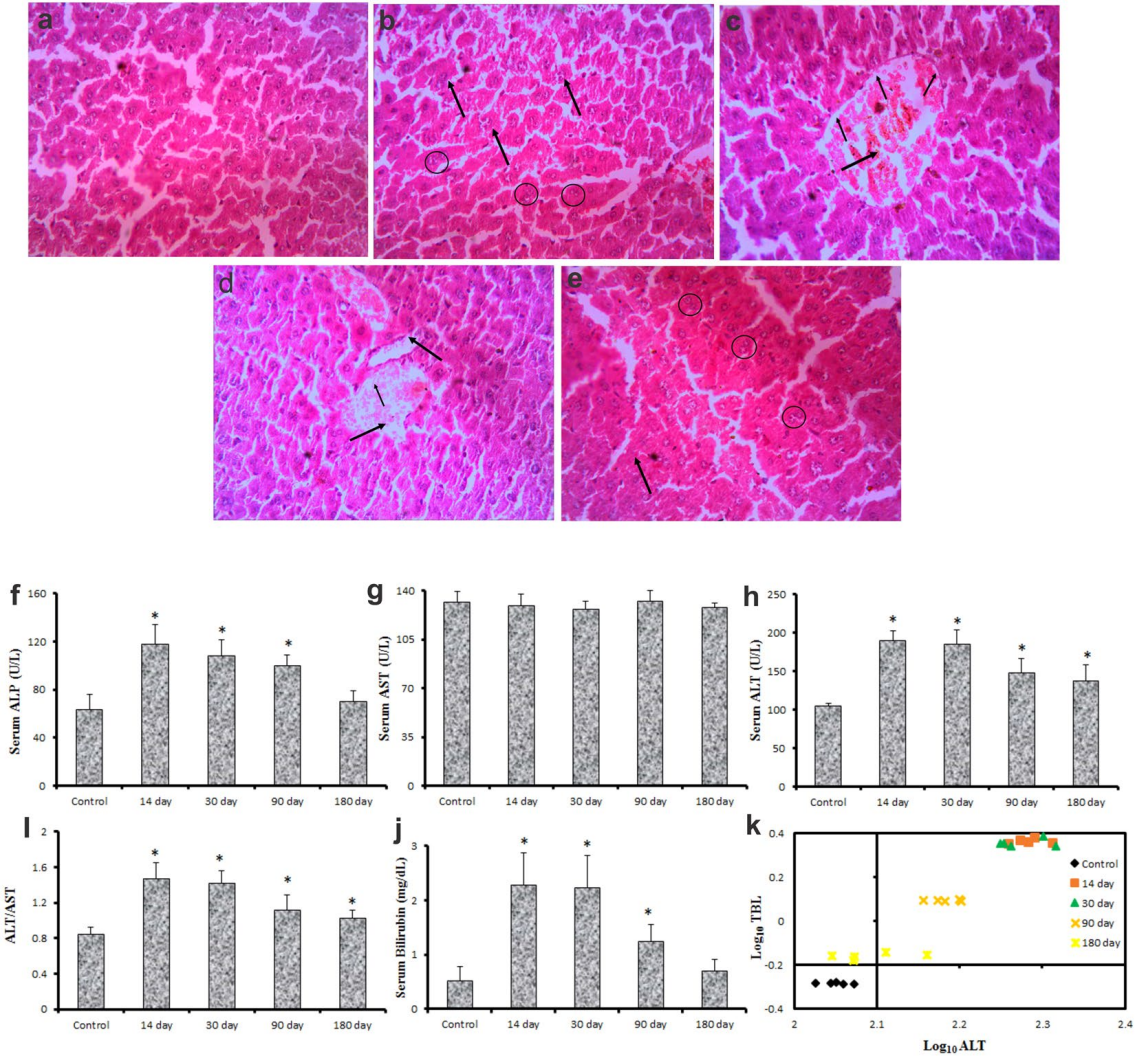
Toxicity profile assessment in liver induced by Bi_2_Se_3_ NP at different time points. (**a–e**) Photomicrograph of representative section of liver excised from (**a**) from control and treated groups on (**b**) 14 day (**c**) 30 day (**d**) 90 day and (**e**) 180 day. Images reveal heaptocyte injury resulted due to disruption of central vein intima, cloudy swelling in hepatocytes (indicate by arrows) and binucleation of hepatocytes (enclosed in circle). (**f–k**) Time course evaluation of hepatic markers indicating cytotoxicity in response to Bi_2_Se_3_ NP exposure. Serum sample from control and treated groups were analyzed for (**f**) ALP (**g**) AST (**h**) ALT (**i**) ALT/AST (**j**) Billirubin (**k**) Zimmerman Concept Log_10_TBL vs Log_10_ ALT. All data are represented as the mean ± SEM (N = 5mice/group) p < 0.05 compared to control group.

The histopathological changes in extrapulmonary organs were also monitored by H&E staining. No apparent histological alterations were observed in kidney and hearts (data not shown), however, Bi_2_Se_3_ NPs induced certain changes in liver that indicated hepatocyte injury (Fig. 7a–e). Hepatocytes exhibited cloudy swelling with pale cytoplasm in all treated groups especially on day 14. The membrane dysfunction with Na^+^ ion and water influx could attribute to hepatocytic ballooning and further result in acute and sub-acute liver injury caused by NP accumulation. Light micrograph images on day 30 and 90 revealed disruption of central vein intima, specifying endothelial damage and vascular stress by NPs. These alterations were also found to be associated with infiltration of inflammatory cells, which could potentially interact with hepatic proteins and enzymes thereby interfering with antioxidant mechanism and leading to ROS generation and further facilitating way for inflammatory responses. Albeit, no kupffer cell hyperplasis was observed, binucleation of hepatocytes was observed in treated groups primarily on day 180 revealing cell regeneration of injured cells. Absence of kupffer cell hyperplasia might indicate low phagocytic activity representing disturbances in defense mechanism causing intoxication.

Markers for hepatic (ALP, AST, ALT, ALT/AST and Total Bilirubin) (Fig. 7f–j) and renal injury (Creatinine, Urea) were examined to evaluate Bi_2_Se_3 _NPs-induced toxicity. ALT and AST are two of the most reliable markers of hepatocellular injury and corresponds well with the extent of liver cell damage^46^. Of the two, ALT is considered more specific as it is mainly found in the cytosol of the liver. The significant increase of ALT at all time points post Bi_2_Se_3 _NP exposure indicated prominent liver injury due to particle accumulation. However, no significant increase was observed in serum AST level. Increase in ALP and TBL was also recorded on day 14 and 30. ALP is primarily detected in the canalicular membrane of the hepatocytes which are the edges of the cells that join to form bile ducts, therefore, blockage of bile ducts resulting in cholestatic problems. Similarly significant elevation in serum bilirubin indicates hepatic cholestatis and loss of liver function^58^.

The changes thus, obtained in biochemical parameters after Bi_2_Se_3_NPs exposure point towards heterogenous liver injury and hepatic cholestatis as well. Therefore, to narrow down the interpretation, we applied the Zimmerman concept; the principle employs plotting ALT vs TBL (in terms of log_10_) over the four quadrants of the graph (Fig. 7k)^58^. Major observations in current study were found to appear in upper right quadrant with elevated ALT and TBL, suggesting hepatocellular damage rather than cholestatic type. The elevation in serum bilirubin level coupled with augmentation in transaminase level indicates severe hepatotoxicity (Hy’s Law). It could, therefore, be predicted that Bi_2_Se_3_ NPs induced liver injury is typically hepatotoxic, however, impairment of bilirubin excretion could not be ignored. Acute liver injury could be attributed either to accumulation or metabolism of Bi_2_Se_3_ NPs. Intratracheal instillation of nanodiamonds induced adverse effect over liver functioning by increasing ALT/AST and ALP expressions^59^. Interestingly, all the markers (except AST) of liver injury were uniformly over expressed on day 14 and 30 post Bi_2_Se_3_ NPs instillation, suggestive of acute liver damage. The enzyme level, however, subsided on day 180 indicative of tissue repair and regeneration. CREA and Urea, important markers of kidney function that is released during muscle metabolism were also measured, however no variation was observed in the levels of the two parameters as compared to the normal (data not shown).

### Outline of metabolism

The immuno-toxicity induced by Bi_2_Se_3_ NPs has been comparatively depicted in the heat map (Fig. 3b). Clustered image maps (CIMs) were observed with the help of CIMMiner program^60^. Assay suits at different time points are represented as per the degree of severity in colors ranging from green to red. Red indicates the maximum while green indicates the minimum value of the data set corresponding to each parameter. The analysis reveals that most of the parameters are on the higher margin on 30 day and 90 day time points displaying severe damage; 180 day also remains to be moderately affected. Peak pulmonary inflammation has been observed in rat lungs at 3 months post intra-tracheal instillation of Nickel oxide NPs^61^. Similarly crystalline silica induced pulmonary inflammation after a latent period of 0–16 weeks post inhalation. Neutrophil counts were elevated at 10 week post exposure rather than on day 4^62,63^. Pertaining to previous findings herein we report that Bi_2_Se_3_ NPs could induce persistent pulmonary inflammation in mice in acute phase and more severely in chronic phase (90/180 days) post exposure, thereby inducing irreversible lesions in lungs^61^.

Based upon the observations of sublethal assay suit, Fig. 3c represents an outline of Bi_2_Se_3_ NPs mediated cell death. Indeed, ROS mediated oxidative stress has been further reported to promote the activation of signaling pathways specifically pro inflammatory cascades. This further initiates cytokine/chemokine expression, toxins stress and DNA damage and regulate immune responses such as apoptosis^64,65^. The present study demonstrates the exaggeration of IL-1β, MIP-2, IL-6 and IL-8 upon nanoparticle internalization leading to increased neutrophil influx and ROS generation. Furthermore, increased expression of cytokines/chemokines could modify protein expression at post translational level, thereby interfering with various transcriptional factors and aggravating additional inflammatory pathways ultimately causing cell death^66,67^.

Albeit intraperitoneally injected PVP coated Bi_2_Se_3_ NPs have been evaluated for their toxicity profile, we for the first time systematically studied the long term immune-toxicokinetics of ~8 nm Bi_2_Se_3_ NPs post intratracheal instillation. Our results reveal severe pulmonary inflammation resulted due to persistence of nanoparticles in lungs. The particle concentration, however, subsided with time but complete clearance could not be achieved. The particles further translocated towards liver indicating involvement of RES in trafficking. The kidney histopathology and biochemistry did not reveal any apparent abnormalities indicating effective particle clearance through renal route. The NPs also induces oxidative damage via ROS generation, which further introduces several pro-inflammatory cytokines resulting in persistent neutrophilic/severe cytotoxic inflammation. However, still a more methodical approach is needed to elucidate Bi_2_Se_3_ NPs-induced cytotoxicity pertaining to their long term accumulation *in-vivo* at different doses. Nevertheless, the results of current studies are highly encouraging and could pave way for future use of Bi_2_Se_3_ NPs in biomedical research.

## Material and Methods

### Synthesis and Characterization of NPs

Bi_2_Se_3_ NPs were synthesized as per sonochemical method^68^. Initially 0.1 mM of cetyltrimethyl ammonium bromide (CTAB) and 0.6 mM of SeCl_4_ were dissolved in distilled water. This solution was continually stirred at room temperature for 30 minutes. Consequently 0.25 g of KBH_4_ was added to the above solution under magnetic stirring to keep the pH value of 12 with NaOH 1.00 M. A solution of 0.4 mM of bismuth and 50 mL of water was made and added in to the above solution drop wise under sonication. After the sonication black precipitates were collected washed with ethanol and distilled water several times and dried in vacuum for 6 hours. Structural properties of these samples were investigated using X-ray diffraction measurement; surface morphology was obtained using scanning electron microscopy. Optical properties were studied using Raman spectroscopy.

X-Ray Diffraction (XRD) measurements were taken using Rigaku Smartlab X-ray Diffractometer, operated at 40 kV/30 mA using CuKα1 radiation with wavelength 0.154 nm in the wide angle region from 10° to 60° on 2θ scale and the phase identification was carried out from standard JCPDS database. The crystallite size d was calculated from the full width of half maximum (FWHM) of the major XRD peak using Scherrer’s formula:

$$d=0.9\lambda /\beta \,{\cos }\,\theta $$

where d is the crystallite size, λ is the wavelength of radiation used, 2θ is the peak position of lattice planes, and β is the full width at half maximum (FWHM) on 2θ scale Raman spectroscopy was executed by using micro-Raman setup with an Ar ion laser beam emitting at a wavelength of 514.5 nm. The spectra were collected for 120 seconds at a power of 10 mW with 1 cm^−1^ spectral resolution. Surface morphology was also observed by scanning electron microscopy (SEM). Images were taken on ZEISS EVO series SEM model EVO15 to find out the size as well as morphology. The surface charge on the NPs dissolved in pure water was determined using a Zeta potential analyzer (Malvern, UK).

### Procurement of Animal model

Eight to ten week-old Balb/c mice were procured and kept under standard animal husbandry conditions; temperature 24 ± 2 °C, relative humidity 50–55%, 12 h light and dark cycle. Following 1 week of acclimatization, mice were randomized into 4 groups (5mice/group) and in order to address the toxicity profile of Bi_2_Se_3_ NPs, a systemic mouse model was developed. All experimental protocols and animal care were carried out in accordance to the guidelines approved by the Institution Animal Ethical Committee (IAEC), University of Allahabad, Allahabad.

### *In-vivo* X-Ray CT imaging

In order to access the dynamic *in-vivo* trafficking of Bi_2_Se_3_ NPs within 60 min, X-Ray CT imaging was performed. First group of mice was administered with Bi_2_Se_3_ NPs intratracheally, while, as control, the second group was administered with the contrast agent Iopamidol. Before administration mice were anesthetized with sodium thiopentone, i.p. 5 mg/0.1 ml/mouse. Mice were scanned by X-Ray CT at 0, 30 and 60 min time points post injection using Scanner (Siemens Healthcare GmbH, Germany).

### Long Term *In-vivo* trafficking

To evaluate the dosage-related pulmonary responses, BALB/c mice were intratracheally instilled with 0, 1, 5 or 10 mg/kg Bi_2_Se_3_ NPs. Animals were exsanguinated on day 7 post exposure. Further, 10 mg/kg body weight of 8 nm Bi_2_Se_3_ NPs were dissolved in MilliQ water and ultrasonicated and vortexed immediately before administering to minimize aggregation of particles. 1 ml of prepared NP suspension was intratracheally instilled into the lungs of mice. Mice treated with saline were referred as control (Fig. 2 Upper panel). To determine long term *in-vivo* distribution of Bi_2_Se_3_ NPs, mice were sacrificed at day 14, 30, 90 and 180, post intratracheal instillation. Tissues were excised from control and treated groups and digested in HNO_3_ and HClO_4_ for 24 h. Digestates were diluted to 10 mL Milli-Q water and Bi_2_Se_3_ particles were analyzed using Inductively Coupled Plasma Mass Spectrometer (ICP-MS) (Thermo Fisher Scientific, Germany). Lung weight and organ weight of control and treated groups were also recorded.

### Bronchoalveolar Lavage Fluid (BALF) analysis for Lung injury and Inflammation

Mice were anesthetized with sodium thiopentone, i.p. 5 mg/0.1 ml/mouse for BALF recovery. The trachea was exposed by midline incision in the neck region 1 ml of PBS was instilled into the lungs through trachea and withdrawn after 10 s. The recovered fluid was centrifuged at 200 rpm for 5 min at 4 °C and the supernatant was utilized for the estimation of LDH (Lactate Dehydrogenase), ALP (Alkaline Phosphatase) and Total protein to evaluate lung injury, cytotoxicity and loss of integrity of alveolar-capillary barriers. Levels of cytokines and chemokines were also measured in BALF supernatant. IL-1β, MIP-2, IL-6, IL-13 and IL-8 were assayed using ELISA kits (R&D System, Minneapolis, Minn., USA).The cells were further fixed in methanol and stained with Giemsa stain. Total cell numbers and different cell types in the BAL fluid were quantified by their characteristic morphologies. Cell death was measured by Propidium Iodide (PI) uptake and flowcytometry^69^. Briefly, cells resuspended in PBS were stained with PI for 15 min in dark. Following incubation cell were analyzed for the fluorescence intensity of PI using Flow cytometer (Becton Dikinson, San Jose, CA, USA).

### Blood biochemical parameter analysis and Histopathology

Blood was collected from retro-orbital sinus with the help of non-heparinized glass capillaries allowed to clot and was centrifuged to separate the serum. Liver function was evaluated by measuring serum levels of alanine aminotransferase (ALT), aspartate aminotransferase (AST), and alkaline Phosphatase (ALP) and Total Bilurubin (TBL). Kidney function was evaluated in terms of Creatinine and Urea. After blood collection mice were sacrificed. Lungs, liver, kidney and heart excised from control and treated mice were fixed in 10% formaldehyde for the assessment of acute and chronic inflammatory responses induced by NP retention. The tissue sections were stained with Hematoxylin and Eosin and observed under microscope.

Lung homogenate was prepared as per Ravichandran *et al*.^70^ with slight modifications. Briefly, whole lung tissue was excised from control and treated groups and washed thoroughly in ice-cold PBS. Tissue homogenate was prepared in 10 mM Tris buffer (pH 7.4) supplemented with protease inhibitor using mortar and pestle followed by sonication. The lysate was centrifuged at 500 g for 10 min at 4 °C. The supernatant was collected and centrifuged again at 2000g for 60 min at 4 °C. The supernatant obtained now was the tissue lysate used further for the estimation of oxidative stress. For the estimation of ROS, 50 μg of protein from each tissue lysate was mixed with 10 μL of 2,7-dichlorofluorescein for 30 min at 37 °C. Following incubation cells were analyzed using Flow cytometer (excitation wavelength 488 nm and emission wavelength at 535 nm).

### Statistical Analysis

The results are presented as the standard error of mean (SEM). A two-way analysis of variance (ANOVA) was used to determine significant (p < 0.05) differences between groups.

## Electronic supplementary material

Supplementary Information
