# Author Correction: Immunotoxicological impact and biodistribution assessment of bismuth selenide (Bi_2_Se_3_) nanoparticles following intratracheal instillation in mice

**DOI:** 10.1038/s41598-020-62927-7

**Published:** 2020-04-07

**Authors:** Vani Mishra, Vikas Baranwal, Rohit K. Mishra, Shivesh Sharma, Bholanath Paul, Avinash C. Pandey

**Affiliations:** 1 Nanotechnology Application Centre (NAC), University of Allahabad, Allahabad, 211002 India; 2 NMR Section, SAIF, CSIR-Central Drug Research Institute (CDRI), Lucknow, 226031 India; 3 Centre for Bioresource Innovation and Research (CBIR), Dept. of Microbiology, Swami Vivekanand University, Sagar, 470228 M.P. India; 4 Centre for Medical Diagnostic and Research (CMDR), Motilal Nehru National Institute of Technology (MNNIT), Allahabad, 211004 India; 5 Immunobiology Division, CSIR-Indian Institute of Toxicology Research (IITR), Lucknow, 226001 India

Correction to: *Scientific Reports* 10.1038/s41598-017-18126-y, published online 21 December 2017

This Article contains errors in Figure 4, where the photomicrographs of representative sections of lungs shown in panels a, c, d and e are incorrect due to misfiling of the data. The correct Figure 4 appears below as Figure 1.

**Figure 1:**
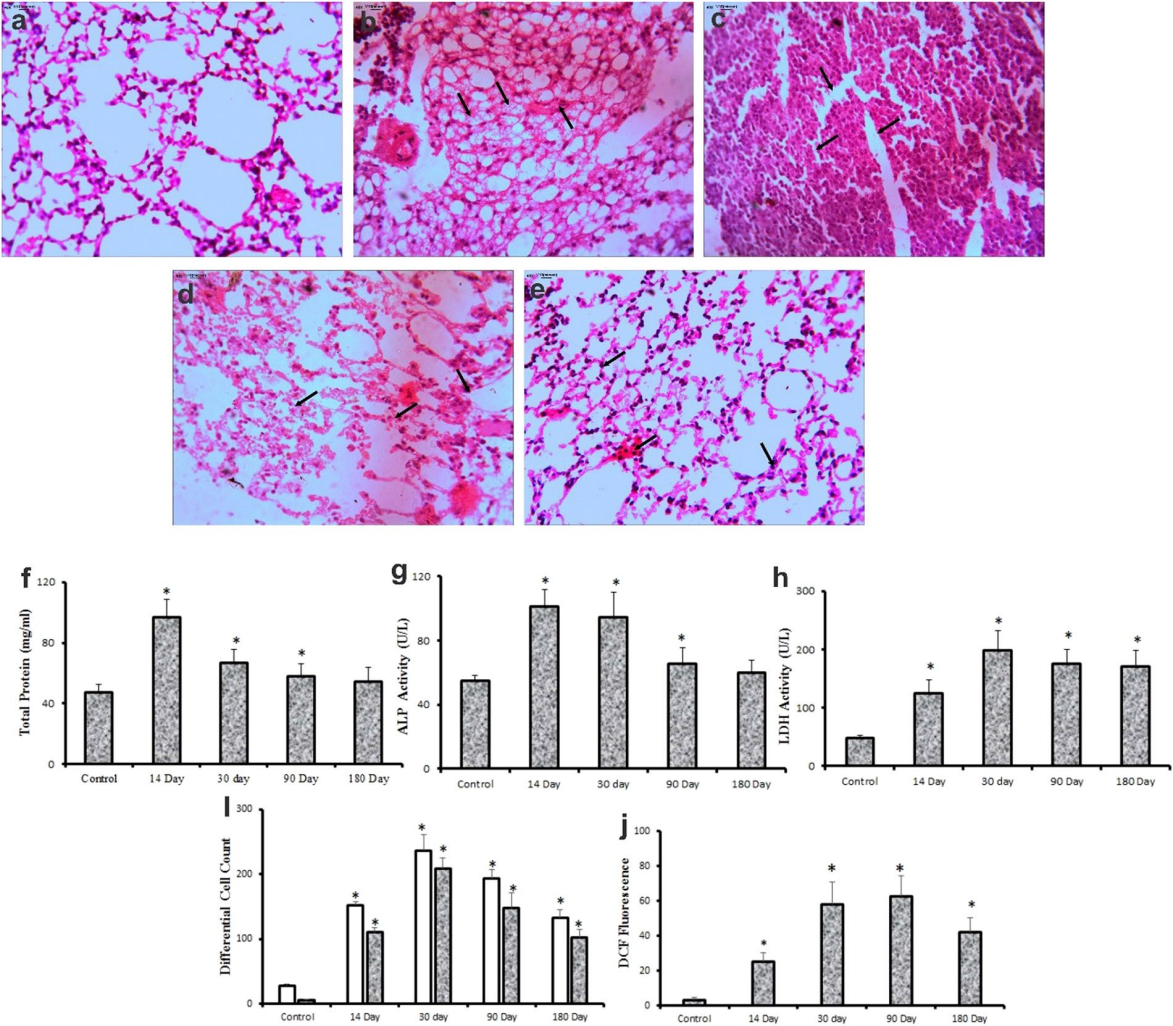
.

