# Supplementary material for: Immunotoxicological impact and biodistribution assessment of bismuth selenide (Bi2Se3) nanoparticles following intratracheal instillation in mice
Source: Sci Rep. 2017 Dec 21;7:18032. doi: 10.1038/s41598-017-18126-y (PMC5740059; doi:10.1038/s41598-017-18126-y)
Supplement: Supplementary file 1 — Supplementary Information [file 41598_2017_18126_MOESM1_ESM.docx]

**Immunotoxicological impact and biodistribution assessment of bismuth selenide (Bi_2_Se_3_) nanoparticles following intratracheal instillation in mice**

*Vani Mishra^1,2*^, Vikas Baranwal^1^, Rohit K. Mishra^3,4*^, Shivesh Sharma^4^, Bholanath Paul^5^,* and *Avinash C. Pandey^1^*

^1^Nanotechnology Application Centre (NAC), University of Allahabad, Allahabad-211002, India.

^2^NMR Section, SAIF, CSIR-Central Drug Research Institute (CDRI), Lucknow-226031, India.

^3^Centre for Bioresource Innovation and Research (CBIR), Dept. of Microbiology, Swami Vivekanand University, Sagar-470228, M.P., India.

^4^Centre for Medical Diagnostic and Research (CMDR), Motilal Nehru National Institute of Technology (MNNIT), Allahabad-211004, India.

^5^Immunobiology Division, CSIR-Indian Institute of Toxicology Research (IITR), Lucknow-226001, India.

***Address of Corresponding authors:**

**Dr. Vani Mishra**

^1^Nanotechnology Application Centre (NAC), University of Allahabad, Allahabad-211002, India.

^2^NMR Section, SAIF, CSIR-Central Drug Research Institute (CDRI), Lucknow-226031, India.

**Email:** [vanimish@gmail.com](mailto:vanimish@gmail.com)

**and**

**Dr. Rohit K. Mishra**

^3^Centre for Bioresource Innovation and Research (CBIR), Dept. of Microbiology, Swami Vivekanand University, Sagar-470228, M.P., India.

^4^Centre for Medical Diagnostic and Research (CMDR), Motilal Nehru National Institute of Technology (MNNIT), Allahabad-211004, India.

**Email:** [rohit_ernet@yahoo.co.in](mailto:rohit_ernet@yahoo.co.in)


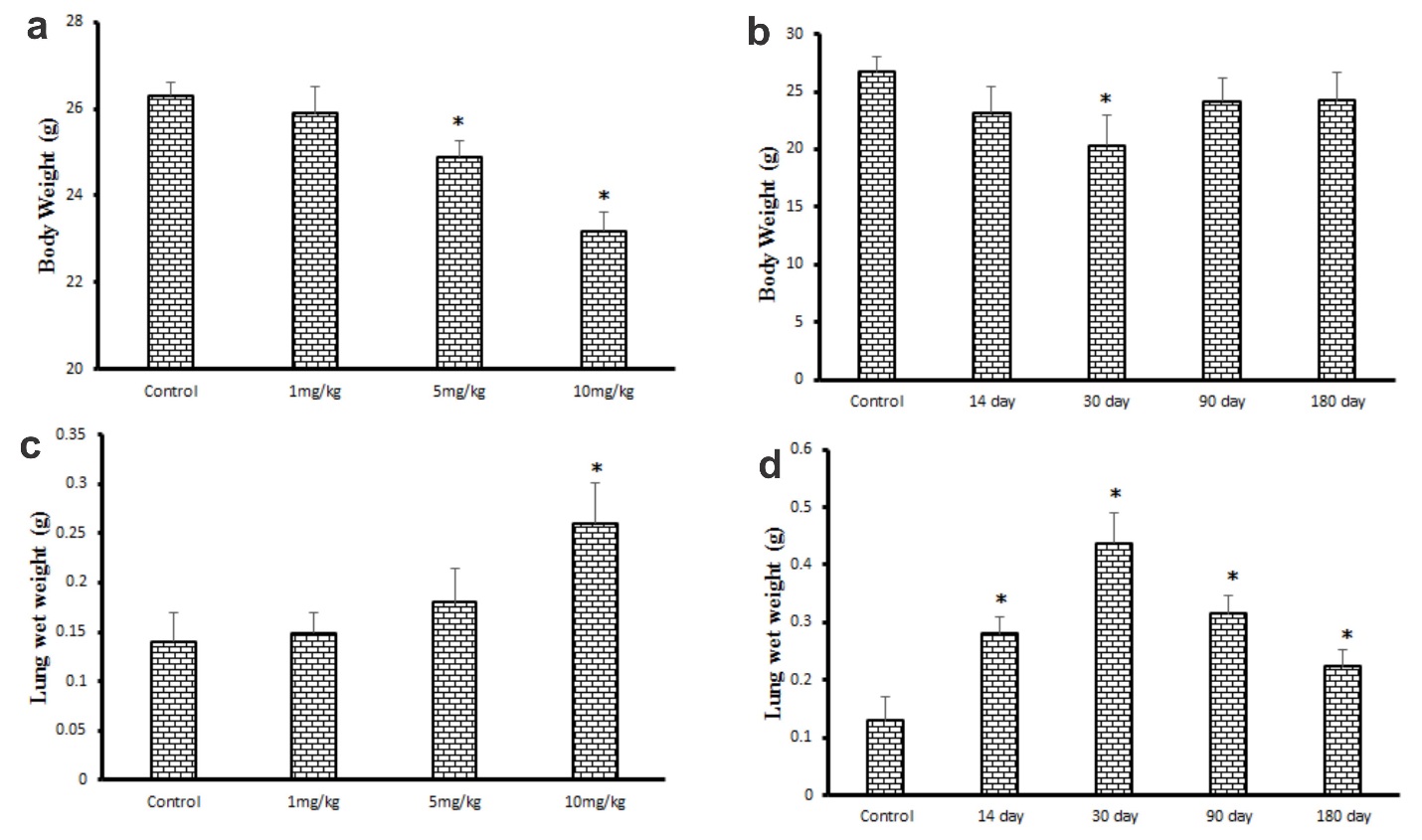


**Figure S1. Evaluation of body weight (a-b), and lung wet weight (c-d).** (**a and c**) represent dose-related response and (**b and d**) represent time related evaluation. All data are represented as the mean ± SEM (N = 5mice/group) p< 0.05 compared to control group.


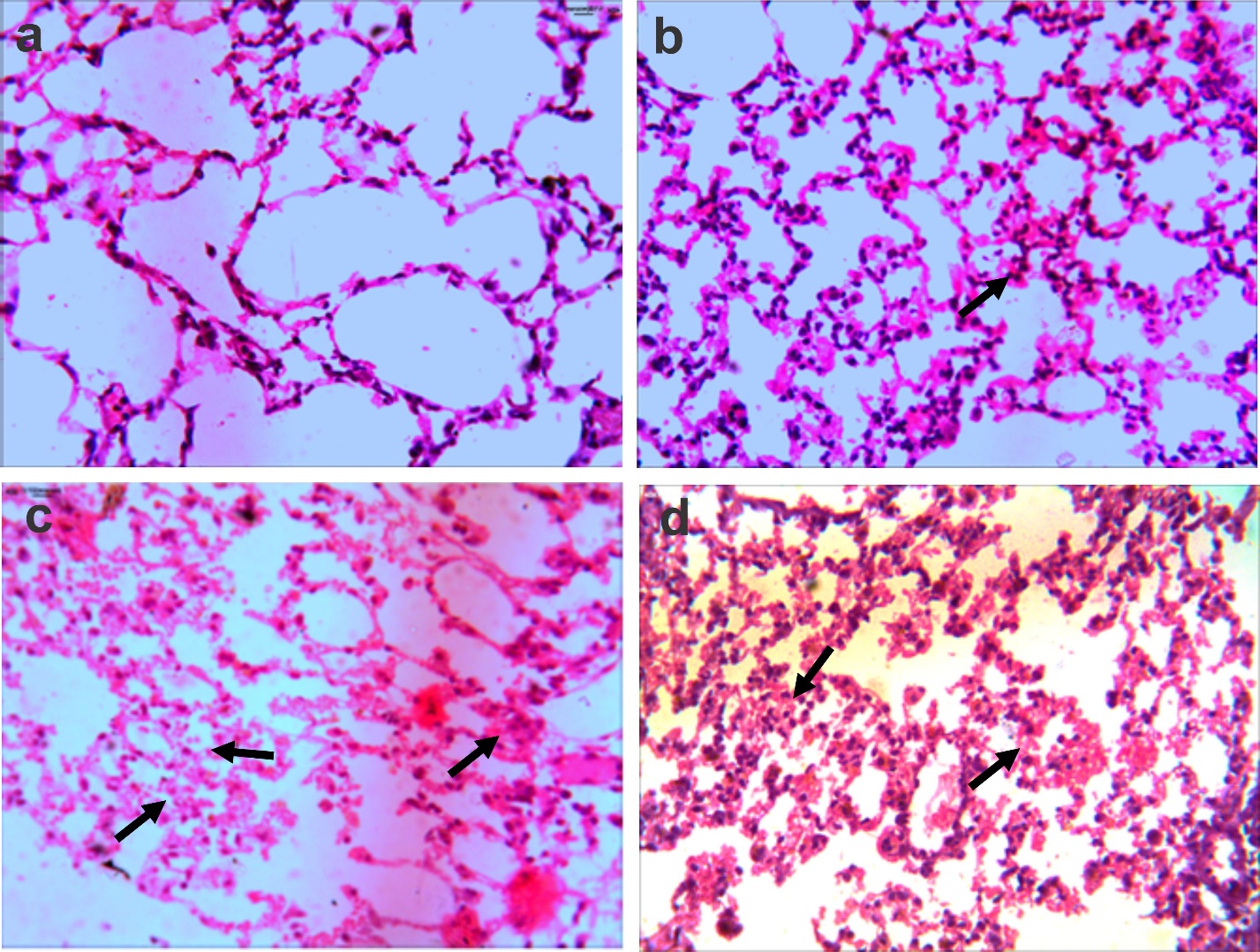


**Figure S2. Histopathological alterations in lungs induced by Bi_2_Se_3_ NP for different doses.** Photomicrograph of representative section of lungs excised from (**a**) from control and treated groups on (**b**) 1mg/kg (**c**) 5mg/kg (**d**) 10mg/kg body weight. Images reveal severe lung damage in treated groups due to infiltration of inflammatory cells, capillary dilation and accumulation of interstitial connective tissues.
